# Supplementary material for: Loss-cone instability modulation due to a magnetohydrodynamic sausage mode oscillation in the solar corona
Source: Nat Commun. 2019 May 23;10:2276. doi: 10.1038/s41467-019-10204-1 (PMC6533292; doi:10.1038/s41467-019-10204-1)
Supplement: Supplementary file 1 — Supplementary Information [file 41467_2019_10204_MOESM1_ESM.pdf]

Supplementary Information

Loss-cone instability modulation due to a magnetohydrodynamic sausage mode oscillation in the solar corona

Eoin P. Carley[1,*], Laura A. Hayes[1,*], Sophie A. Murray[1,*], Diana E. Morosan[2,1], Warren Shelley[1], Nicole Vilmer[3,4] & Peter T. Gallagher[1,*]

[1] *Astrophysics Research Group, School of Physics, Trinity College Dublin, Dublin 2, Ireland.*

[2] *Department of Physics, University of Helsinki, P.O. Box 64, Helsinki, Finland.*

[3] *LESIA, Observatoire de Paris, PSL Research University, CNRS, Sorbonne Universités, UPMC Univ. Paris 06, Univ. Paris Diderot, Sorbonne Paris Cité, 5 place Jules Janssen, 92195 Meudon, France.*

[4] *Station de Radioastronomie de Nançay, Observatoire de Paris, PSL Research University, CNRS, Univ. Orléans, 18330 Nançay, France.*

**Current address: Astronomy & Astrophysics Section, School of Cosmic Physics, Dublin Institute for Advanced Studies, 31 Fitzwilliam Place, Dublin. (email: eoin.carley@dias.ie)*

Supplementary Note 1: Metric radio and EUV observations throughout the event

Supplementary Figure 1a shows the initial type III burst at the start of the event at approximately 12:51 UT, while Supplementary Figure 1b shows the location of the pulsation source, and Supplementary Figure 1c shows an EUV wave propagating across the quiet AR in an easterly direction. This wave is the likely source of the disturbance of the coronal loops, leading to the radio activity in the quiet AR.

Supplementary Figure1c-f, and h show a complete overview of the NRH flux density from 150–445 MHz at the site of the pulsations throughout the event. Radio emission begins to rise in all frequencies at approximately 12:45 UT. This initial rise is the first evidence of energetic electrons occurring specifically in the quiet AR, followed by the type III radio burst at 12:51 UT, observed at 150–228 MHz (also indicated in Figure 2 of the main article). The slow rise in activity can also be seen in the extreme ultraviolet (EUV) as a rise in flux in AIA 9.4 nm and 13.1 nm filters in Supplementary Figure 2, which indicates heating in this region (due to small levels of flaring and electron acceleration). Supplementary Figure 3 below also indicates an increase in hard X-ray flux

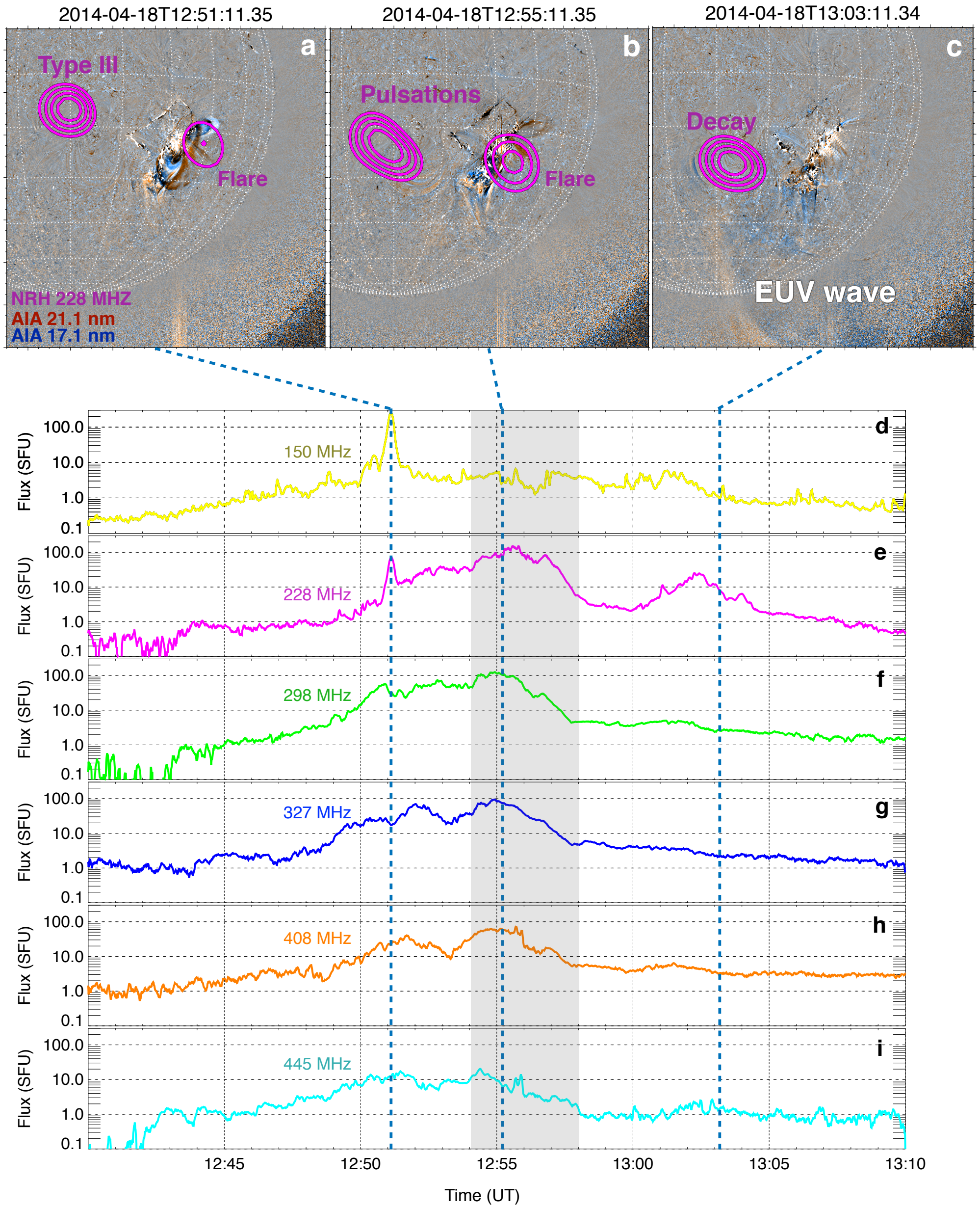

Figure 1: Metric radio emission before during and after the pulsation source. (a) AIA two color running ratio image composed 17.1 and 21.1 nm, overplotted with NRH 228 MHz contours showing the position of the initial type III radio burst (at 12:51 UT in the dynamic spectrum of Figure 2, main article). (b) Positions of the pulsations for comparison. (c) A faint EUV wave is visible passing over the active region. A decay source is present at this time, showing there is ongoing electron energisation. Panels d-i show a complete overview of the NRH flux density from 150–445 MHz at the site of the pulsations throughout the event. The dashed lines indicate the image times, while the shaded grey area shows the duration of the pulsations.

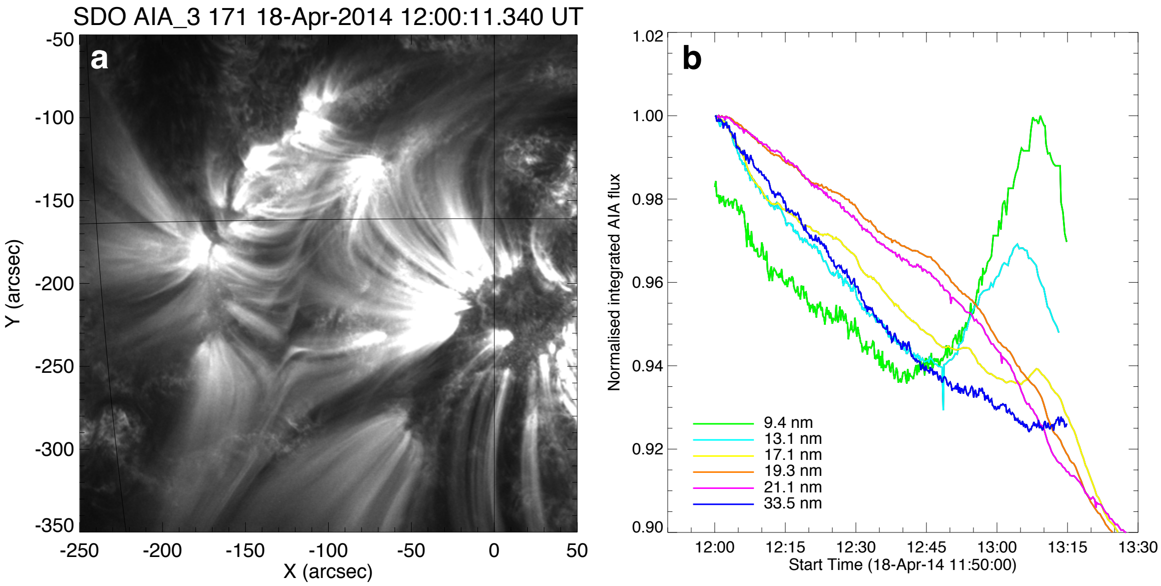

Figure 2: EUV activity taking place at the site of the pulsations. (a) The pulsation site images in AIA 17.1 nm. As a measure of activity at this site, the total EUV flux was integrated over this region in the EUV channels of AIA. (b) Normalised integrated flux in AIA channels (indicated by color), showing a rise in flux in 9.4 nm and 13.1 nm. The rise in flux from these channels is an indicator that some level of plasma heating took place from 12:45 UT, coinciding in time with the initial rise in metric radio flux seen in Supplementary Figure 1 and rise in HXR and microwave flux seen in Supplementary Figure 3 below. This is an indicator of some initial electron acceleration and plasma heating taking place before the pulsations begin.

(HXR) and microwave flux from approximately 12:45 UT onwards). It is not clear what initially causes this slow rise in activity from 12:45 UT onwards, but may be due to the quiet AR being perturbed by an EUV wave during the eruption of the flare AR at this time.

The initial activity is followed by the radio pulsations, shown by the shaded grey region in Supplementary Figure 1a. The type III and pulsation sources occur at similar locations and likely had a similar origin e.g., when the active region becomes initial perturbed, there is an initial electron energisation (observed via increases in metric radio, microwave, HXR and EUV flux). Note that Supplementary Figure 1c is after the pulsation event, but there is still some on-going radio sources (electron energisation) as the EUV wave passes (the radio source is labelled decay source here because it occurs in the final stages of the event and it is not associated with any

significant activity in the dynamic spectrum). The radio emission diminishes at all frequencies when the EUV wave has passed completely.

Supplementary Note 2: Pulsation association with HXR and microwave observations

The rise in radio and EUV activity is an indicator of electron acceleration taking place before and during the pulsations. Inspection of the pulsation details in the dynamic spectra in Figure 4 of the main article also reveals the presence of reverse drift bursts. These bursts have morphology and a drift rate (approximately 220 MHz $^{-1}$) that are similar to the previous observations of reverse drift bursts that were attributed to electron beam propagation. Furthermore, using the density model constructed from the DEM analysis we derived an exciter speed for the reverse drifters of 0.2 c. This is good evidence to suggest that electron beams are involved in the excitation of the pulsations.

To confirm the presence of energetic electrons during the pulsations we show the relationship between the radio flux from pulsations at 228 MHz from NRH alongside HXR curves from FERMI Gamma Ray Burst Monitor [GBM; 1] and also microwave flux from the Radio Solar Telescope Network [RSTN; 2], San-Vito site. The HXR lightcurves indicate photon energies of 4.5-11.8 keV, 11.8-26.9 keV, 26.9-50.4 keV and 50.4-101.6 keV, see Supplementary Figure 3b, while the microwave flux is shown for 2.4, 4.9, 8.8, and 15.5 GHz. Note the initial rise in flux at 12:45 UT observed in metric radio and EUV is also observed in HXR and microwave.

We have indicated five peaks in the HXR data labelled p1-5 in Supplementary Figure 3c. Peak p1 corresponds to the initial type III from the quiet AR as discussed above; it is observed in both microwave and HXR, which is a good indicator that the energy release that accelerated electrons to produce type IIIs also produce downward propagating electrons to produce microwave (gyrosynchrotron) and HXR emission (thick-target bremstrahlung). Peaks p2 and p3 are observed in HXR but show no corresponding peaks in the radio lightcurve, however we do not expect every peak in HXR to have a corresponding plasma emission. Peaks p4 and p5 again are observed in both HXR, metric radio and microwave. The details of the final two peaks p4 and p5 are shown in Supplementary Figure 3d along with the flux of the pulsating radio source at 228 MHz and the 4.9 GHz flux from RSTN. Interestingly, the long time-scale variation of the NRH pulsation flux, the HXR flux at 50-100 keV and the microwave flux follow each other closely from 12:55 UT to

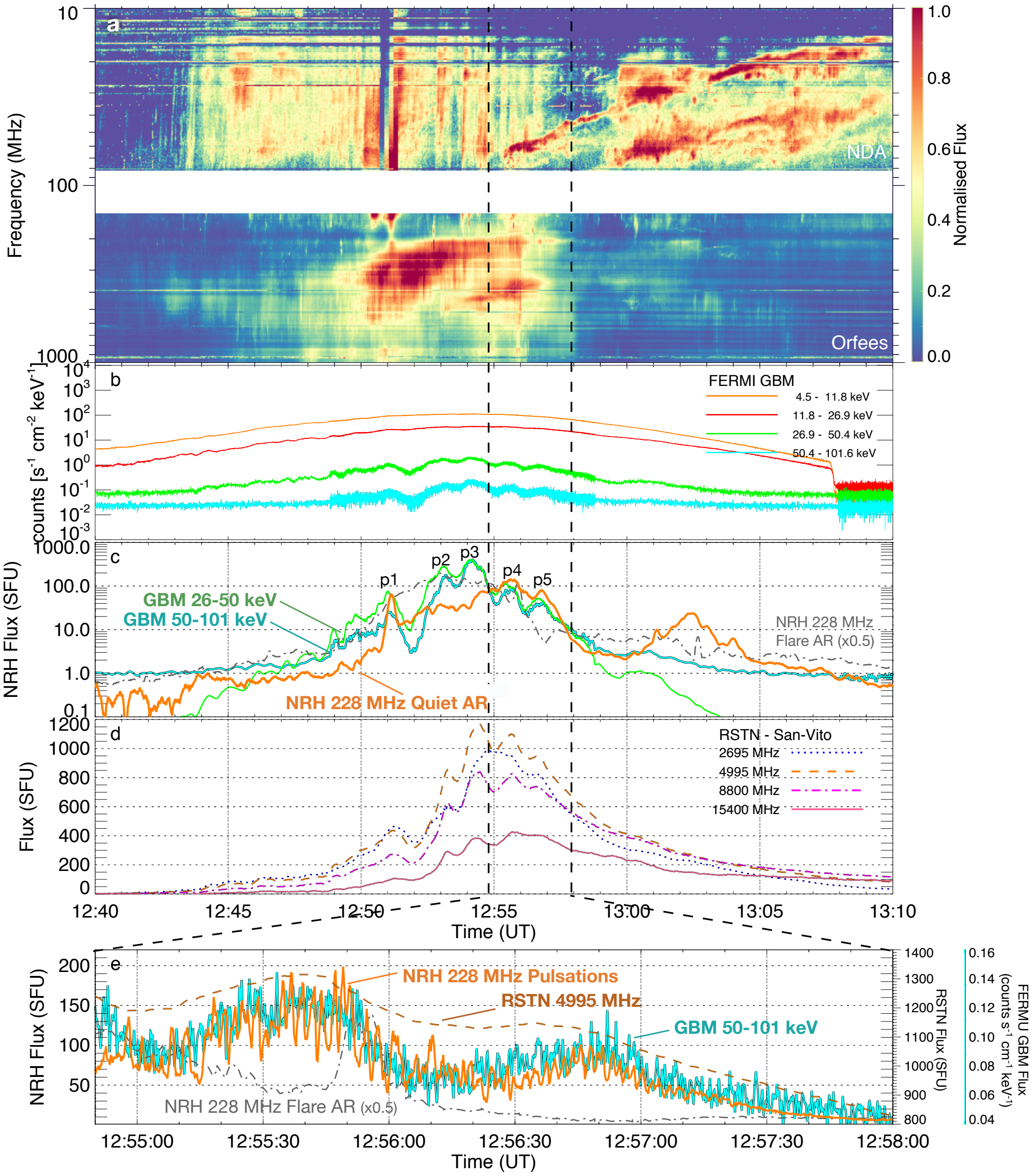

Figure 3: Relationship of pulsating metric radio source to HXR and microwave data. (a) Dynamic spectra from NDA and Orfées, (b) HXR from FERMI GBM showing a slow rise and fall during the event. (c) A comparison of HXR 26-50 and 50-101 keV (scaled arbitrarily) to the NRH 228 MHz flux from the quiet AR. Five peaks p1-p5 are indicated. (d) Single frequency flux from RSTN San-Vito. (e) A zoom of peaks p4 and p5 at the time of the pulsations, comparing NRH 228 MHz, GBM 50-101 keV (26-50 keV shows the same behaviour) and RSTN at 8.8 GHz (flux values of RSTN and GBM shown on the right-hand axes). The curves follow each other closely, showing that the pulsations were associated with HXR and microwave flux. During the pulsation time, the radio flux from the flare AR (shown by the grey dot-dash line) shows little comparison to the activity in the quiet AR.

12:58 UT. This is further evidence that the pulsation source was closely associated with energetic electrons. The electrons responsible for the pulsing emission (and sudden reductions) are related to those causing the HXR and microwave e.g., the accelerated electrons which cause the metric radio would continue to low altitudes in the corona. While preciptating to lower altitudes these electrons emit gyrosynchrotron and eventually reach the chromosphere and emit HXR through the usual thick target mechanism, similar to the findings of [3] and [4]. Unfortunately, at timescales of the pulsations (approximately 2 seconds) the GBM and microwave light curves show no indicating of pulsations and display only noise. For example, during the pulsation event from 10:55-10:58 UT GBM 50-101 keV reaches a max photon count of approximately 600 (counts integrated over time, energy and detector area). Poisson noise at such a photon count is $\sqrt{600} = 24$. The variability in the GBM counts on timescales of seconds is of the same order as this Poisson noise, so the electrons accelerated during the pulsations do not have an observable X-ray signature on timescales of seconds.

Finally, given the simultaneous increase in radio activity in the quiet and flare AR, it may be suggested that electrons originally accelerated at the flare site had access to the pulsations site. However, Supplementary Figure 3d shows the flare AR radio source shows little correlation with the pulsations, HXR and microwave emission from the quiet AR. Hence electrons accelerated during pulsations do not originate from the adjacent flare site.

Supplementary Note 3: Pulsation activity in separate Q and P sources

Supplementary Figure 4a shows the NLFFF magnetic field extrapolation with NRH 228 (red), 298 (green) and 327 (blue) MHz radio sources. For the 228 MHz source, the fluxes at P and Q (on either side of the black dotted line) are treated separately, shown in Supplementary Figure 4b. This shows the pulses primarily come from point P around the magnetic null point, while radio flux at Q shows steadier variation in intensity and smaller levels of pulsation. The higher frequencies of 298 and 327 MHz, are mainly concentrated around point Q (yellow region). Their fluxes in Supplementary Figure 4c show a smoother variability, with little sign of pulsation. Sources of 408, 432 and 445 MHz from NRH have the same position at Q (not shown in the figure), with their fluxes shown in Figure 4c (they behave much the same as the 298-327 MHz sources, but the

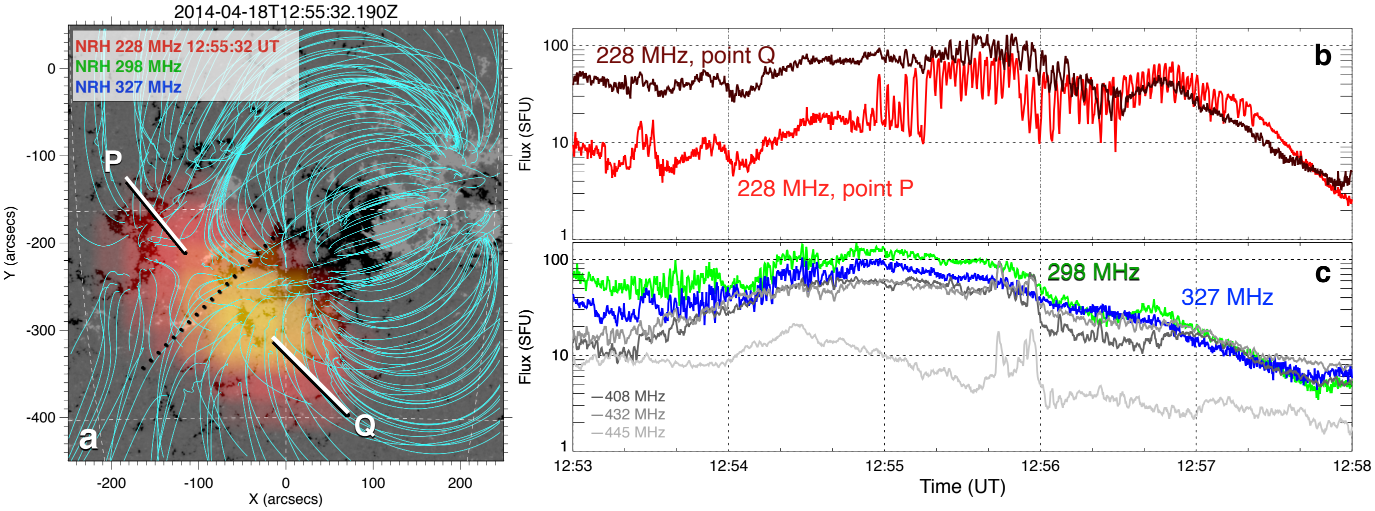

Figure 4: Flux density of separate P and Q sources. (a) NLFFF magnetic field extrapolation is overlaid on HMI magnetogram, with NRH 228 (red), 298 (green) and 327 (blue) MHz radio sources. From supplementary movie 1, two 228 MHz radio sources are identifiable around points P and Q. Fluxes of these sources are calculated separately (flux calculated on either side of the dotted black line). These separate fluxes are shown in panel b, revealing that the primary source of the radio pulses is at point P, while the source at Q shows a smaller amount of pulsation with a steadier variation in intensity. The higher frequencies of 298 and 327 MHz concentrate around point Q and show a steady variation in intensity (panel c), with minimal pulsation signature. The 408, 432 and 445 MHz sources (not shown in images) have the same positions around Q with their light-curves shown in panel c; their behaviour is much the same as the 298-327 MHz emission, with the 445 MHz being the weakest in flux density.

445 MHz source is quite weak). Overall, there is a pulsation of radio intensity at 228 MHz at point P, while 228–432 MHz radiation at Q is more smoothly varying.

Supplementary Figure 5 shows the radio emission from 228-327 MHz is right hand circularly polarised (negative Stokes V), at approximately 20–70%. Negative circular polarisation in a negative magnetic field (as seen in the HMI magnetogram) means this radiation is X-mode polarised.

Supplementary Note 4: Apparent motion of the pulsating radio source

Supplementary Figure 6a shows the locations of the radio sources (sites of energised electrons)

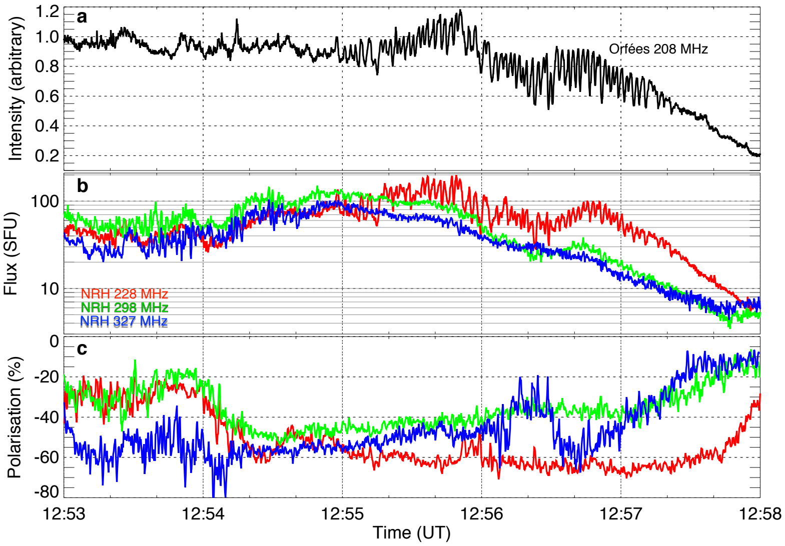

Figure 5: Polarization characteristics of the pulsating radio source. (a) Pulsation radio sources intensity from Orfées at 208 MHz. (b) Pulsation radio source intensity at NRH 228 MHz (red), 298 MHz (blue) and 327 MHz (green). (c) The Stokes V/I polarisation of the radio sources.

over time at frequencies from 228-327 MHz, overplotted on AIA 17.1 nm. The coloured points show the 228 MHz (red), 298 MHz (green) and 327 MHz (blue) source peak positions every 0.25 seconds from 12:55 UT to 12:58 UT (progression through this time is represented by light-to-dark shading). The average spatial positions of each cluster of points is marked by a circle, with the 228 MHz average spatial position labelled R. Firstly, the 228 MHz emission is concentrated in a narrow band from Q to P. There is an overall light-to-dark shading of the maximum emission position from Q to P, indicating a source maximum position shift towards the east over a timeframe of approximately 4 minutes. This general displacement from Q to P over the lifetime of the burst can be seen by the displacement curve in Supplementary Figure 6b. This plot also shows the ratio of the flux densities of sources at Q and P. Comparison of the Q/P ratio and displacement curve shows that the displacement of the source over the course of the event is due to a decreasing Q/P ratio i.e., due to the source at P becoming relatively brighter over time. This also applies to the finer time-scale motion of the radio sources. Supplementary Figure 6c shows that during each pulse at P the flux ratio Q/P drops, resulting in a displacement towards P. Hence this analysis shows that there are two components of radio source max position change, the gradual shift eastwards superimposed by the back-forth motion during the pulses (evident in Supplementary Movie 1). These motions are

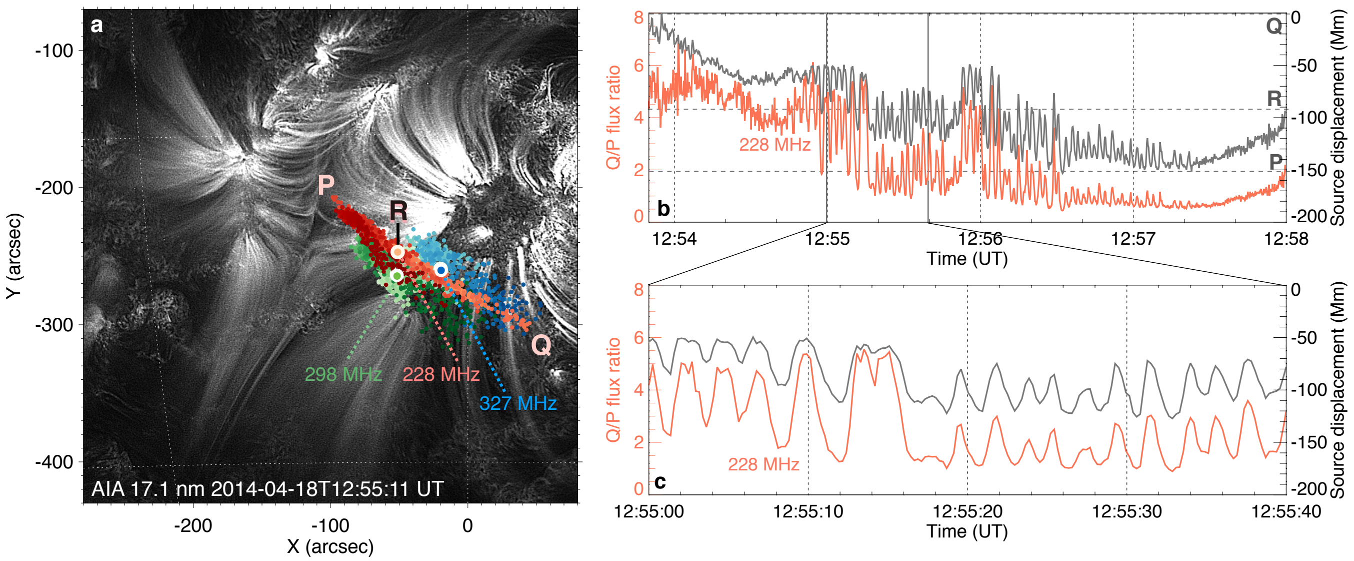

Figure 6: Radio source positions throughout the pulsation source lifetime. (a) The positions of all radio source maxima over time are shown by the coloured points, with NRH 228 MHz (red), 298 MHz (green), 327 MHz (blue); the circles mark the average positions of these point clusters, with the 228 MHz source average position marked by R. (b) Ratio of flux densities of the 228 MHz sources at Q and P (red) and the displacement of the 228 MHz radio source maximum. The Q/P ratio and source displacement follow each other closely, both for the long time-scale displacement from Q to P and for the finer time-scale motion during the pulsations shown in panel c. This shows that any source motion during the pulsation event is apparent motion and due to the alternating brightnesses of sources at Q and P.

only apparent, due to the slow growth in the P-source relative brightness (leading to gradual shift eastwards) and alternating relative brightness of P and Q sources during the pulsations (leading to the back-forth finer timescale motion). That said, during intensity pulses there are signatures of electron beams in the dynamic spectrum, indicating that there may be some beam propagation away from the pulsing source at P. Because the separation of the two radio sources at P and Q is so close to the resolution limit of NRH at 228 MHz (100″), it is difficult to distinguish between actual radio source motion (due to beam propagation) and the apparent motion caused by the alternating brightness of the two sources.

For the 298 and 327 MHz sources, there is no such periodic motion. As shown by the green

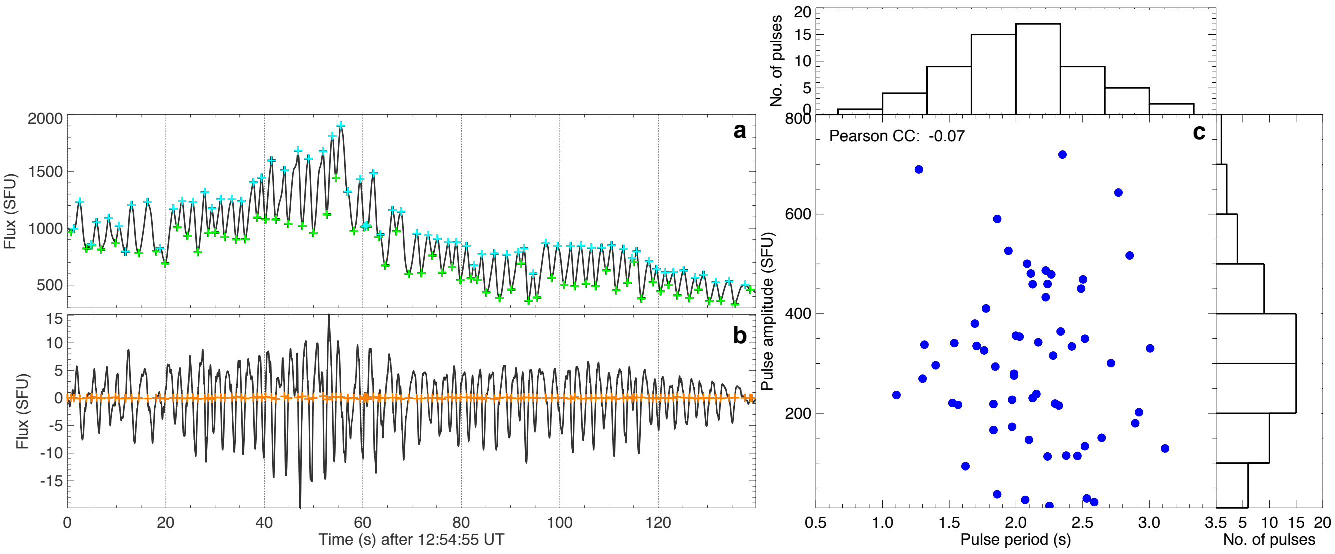

Figure 7: Pulsation flux and period analysis. (a) Total flux (no background subtraction) of Orfées at 208 MHz versus time with pulse maxima (blue crosses) and minima (green crosses) indicated. (b) Time derivative of Orfées at 208 MHz total flux. The maxima and minima in panel a are found using the times at which the pulse profile derivative is zero. (c) Pulse amplitude versus pulse period for all pulsations, showing no correlation.

and blue points in Supplementary Figure 6a, they are scattered around points R to Q and remain relatively steady in position. This shows that there is pulsing energisation of electrons at P and simultaneously a concentration of electrons in the region between P and Q.

Supplementary Note 5: Likelihood of a Lotka-Volterra system

Although radio emission modulation from an MHD oscillation is a likely scenario, we cannot rule out other mechanisms leading to the radio pulsations. The other primary candidate in the production of radio pulsation is known as a Lotka-Volterra or a predator-prey system. In this mechanism a wave energy distribution $N(\mathbf{k})$ (where $\mathbf{k}$ is wavenumber) and electron distribution $f(\mathbf{p})$ ($\mathbf{p}$ is momentum) periodically exchange energy. The linearised Lotka-Volterra system has well-known solutions, the shape of which can easily resemble the sawtooth pattern we observe here, with the period given by $\tau_{\text{pulsations}} = \sqrt{\tau_{\text{growth}}\tau_{\text{diff}}}$ where τ_{growth} is the wave energy growth time and τ_{diff} is the electron diffusion time . However, such a mechanism is very sensitive to any non-linear effects. For example, even minor perturbations of the electron energy distribution

would result in an exponential increase in the wave energy growth rate [5]. Such non-linear effects in the LV system should produce the relationship $F \sim \tau_{\rm pulse}^2$ [6, 7], where F is pulse flux and τ is pulse period. Here we specifically test for such a relationship. Supplementary Figure 7a shows an analysis of the pulse periods and flux amplitudes. The pulse maxima and minima found using a peak finding algorithm that uses the times at which the pulse profile derivative is zero (shown in Supplementary Figure 7b). For a single pulse the amplitude is taken to be the distance from a green to the next blue point (minimum to maximum), while the pulse period is the distance between the green points (minimum to minimum). The relationship between pulse amplitude and period for all pulses is shown in Supplementary Figure 7c, along with histograms for each property. We find that no $F \sim \tau_{\rm pulse}^2$ relationship exists between pulse flux amplitude and period for this event. Since these properties are uncorrelated, and pulse period and amplitudes are regular with no non-linear perturbations, we consider the LV-system to be less likely in the explanation of the radio pulsations.

Supplementary Note 6: Pulsation emission mechanism and dominant ECM modes

ECM instabilities generally result in three principle wave modes in the plasma, namely the O, X and Z mode [8, 9, 10, 11, 12, 13]. Growth of the Z-mode is possible in regions of the corona where $\omega_{\rm e}/\Omega_{\rm e} > 1$, where $\omega_{\rm e}$ is electron plasma frequency and $\Omega_{\rm e}$ is electron cyclotron frequency. In this event the emission frequency of 228 MHz occurs at >13 Mm altitude (assuming fundamental emission). The magnetic field strength at such an altitude is approximately 50 G in the NLFFF analysis, resulting in a cyclotron frequency of 140 MHz and a ratio of $\omega_{\rm e}/\Omega_{\rm e} = 1.6$ i.e., Z-mode favourable conditions (if harmonic emission is assumed the height changes to approximately 50 Mm, which results in a ratio of $\omega_{\rm e}/\Omega_{\rm e} = 2$, which is again Z-mode favourable). Furthermore, electromagnetic emission from Langmuir waves generated from a loss-cone distribution is expected to be X-mode polarised for harmonic emission [14, 15, 16]. Supplementary Figure 5 above shows the radiation in this event is X-mode circularly polarised up to 70%.

In total, the characteristics of the pulsation emission are narrow bandwidth (30 MHz), high intensity (approximately 900 SFU), X-mode polarisation ($\leq$70% -V), extreme spectral slopes ($\alpha_+ = 21$, $\alpha_- = -13$), along with Z-mode conditions of $\omega_{\rm e}/\Omega_{\rm e} = 1.6$ or $\omega_{\rm e}/\Omega_{\rm e} = 2$, depending on the harmonic. All of these characteristics are consistent with the behaviour we would expect of plasma

emission generated from a loss-cone instability. We also showed in Figure 3b of the main article that emission from 228-327 MHz is primarily concentrated around the coronal loop footpoints and is related to higher magnetic field strengths, where we would expect a loss cone to occur. Hence there is a variety of evidence in the form of both spectral characteristics and emission source location that are in support of the ECM Z-mode being involved in the pulsation mechanism.

On the other hand, the O and X mode have high growth rates at $\omega_\mathrm{e}/\Omega_\mathrm{e} < 1$ (although exact ratios at which the modes dominate depend on the electron distribution function [17, 18]), and lead to direct emission from the plasma at frequencies close to the cyclotron frequency. In this event, X or O modes occurring at 228 MHz would require a magnetic field strength of 81 G, which occurs $<$10 Mm in the NLFFF extrapolation (see Figure 5c of main article). Densities at such low altitude are likely to be quite high at $>10^9$ cm^{-3} (from the density diagnostics provided from the emission measure analysis), which would result in $\omega_\mathrm{e}/\Omega_\mathrm{e} \approx 1.6$. The majority of studies determine this ratio to be unfavourable to the production of the O and X modes [5, 12, 17], however some authors have shown such modes can grow to observable levels with $\omega_\mathrm{e}/\Omega_\mathrm{e} > 2$ [18]. In this case, we consider the Z-mode to be more likely in our case due to the large ratio of $\omega_\mathrm{e}/\Omega_\mathrm{e}$, but also due to variety of emission characteristics stated above.

Supplementary Note 7: Viability of the sudden reduction mechanism

Similar to the analysis of [19], the pulsations in this case may be due to sudden interruption of a loss-cone mechanism due to new injection of electrons into a magnetic trap in which the ECM is at play. This leads to sudden reductions in the emission, as shown in Supplementary Figure 8. For the sudden reduction mechanism to be viable the pulse decay time must follow $t_\mathrm{decay} = t_\mathrm{inj} + t_\mathrm{damp} \lesssim 0.9$ (average pulse decay time), where t_inj is the time taken for electron injection that quenches the loss-cone instability and t_damp is the time taken for collisional damping of the Langmuir waves that cause the emission. The pulse rise time would be $t_\mathrm{rise} = t_\mathrm{lc} + t_\gamma \lesssim 1.1$ (average pulse rise time), where t_lc is the time taken for electrons within the loss cone to escape the trap and t_γ is the growth time of plasma waves that cause the emission recovery. The pulse period would then be $t_\mathrm{pulse} = t_\mathrm{rise} + t_\mathrm{decay}$. Firstly, the damping time of the Langmuir waves is approximately equal to the inverse of the collision time of thermal electrons with ions, given by $t_\mathrm{damp} = T^{3/2}/(5.5n\ln 10^4 T^{2/3} n^{-1/3})$

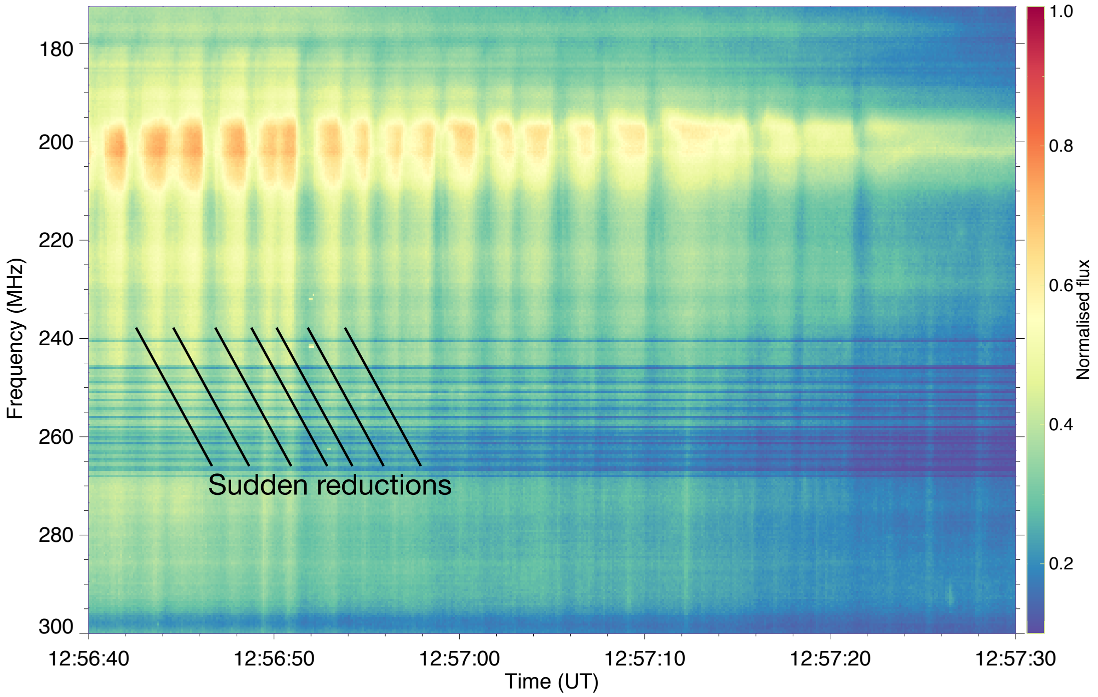

Figure 8: Orfées observations of sudden broadband reductions in the pulsation dynamic spectrum. These resemble the sudden reduction features studied in [19] and are attributed to quenching of a loss cone instability due to injection of electrons into a magnetic trap.

[19], where T is temperature and n is the electron density. Taking $T \approx 10^6\,\mathrm{K}$ and $n \approx 10^9\,\mathrm{cm}^{-3}$, $t_{damp} = 15\,\mathrm{ms}$. This limits the injection time of the electrons to being $\lesssim 0.9$ second, which is the approximate time of the reverse-drift beam signature. This tells us that once the beam is injected and loss-cone quenched, the resonant waves which are causing the emission decay rapidly, resulting in the sudden reduction. For the rise phase, we use the plasma wave growth time of $t_\gamma < 0.2\,\mathrm{s}$ from [19]. This results then in the remainder of the rise being due to $t_{lc} = L\tan\alpha/u < 0.9\,\mathrm{s}$, where L is the trap length scale, α is the loss-cone half-angle and u is the phase speed of the waves which grow due to the instability (also the perpendicular speed of the electrons which cause the resonance of waves at phase speed u). Given $t_{\mathrm{lc}} \lesssim 0.9\,\mathrm{s}$, $L \leq 100\,\mathrm{Mm}$ (approximate distance between P and Q) and $v_{\mathrm{e}} \leq 0.2\,\mathrm{c}$ (speed calculated from drift rate of the reverse drift bursts), the resulting loss cone half-angle is $\alpha \leq 30°$. Such a loss-cone angle would occur if the injection point of the electrons had a field strength of $B_0 \leq 0.25 B_{\mathrm{m}}$, where B_{m} is the field strength at the mirror point. In Figure 5c in the main article, the electrons are accelerated close

to an environment of approximately 50 G, meaning the mirror point would have a field strength of 200 G. From the NLFFF extrapolation, such field strengths occur after a relatively short distance, hence explaining the proximity of P and Q. Given each characteristic time calculated above for injection and loss-cone instability quenching, wave decay, loss-cone reformation and plasma wave growth, the sudden reduction mechanism is viable on the timescales of growth and decay in the pulsations in this event.

References

1. Meegan, C. *et al.* The Fermi Gamma-ray Burst Monitor. *Astrophysical Journal* **702**, 791–804 (2009). `0908.0450`.

2. Guidice, D. A. Sagamore Hill Radio Observatory, Air Force Geophysics Laboratory, Hanscom Air Force Base, Massachusetts 01731. Report. In *Bulletin of the American Astronomical Society*, vol. 11, 311–312 (1979).

3. Aschwanden, M. J., Benz, A. O. & Schwartz, R. A. The Timing of Electron Beam Signatures in Hard X-Ray and Radio: Solar Flare Observations by BATSE/Compton Gamma-Ray Observatory and PHOENIX. *Astrophysical Journal* **417**, 790 (1993).

4. Kupriyanova, E. G., Kashapova, L. K., Reid, H. A. S. & Myagkova, I. N. Relationship of Type III Radio Bursts with Quasi-periodic Pulsations in a Solar Flare. *Solar Physics* **291**, 3427–3438 (2016). `1608.00129`.

5. Aschwanden, M. J. & Benz, A. O. On the electron-cyclotron maser instability. I - Quasi-linear diffusion in the loss cone. II - Pulsations in the quasi-stationary state. *Astrophysical Journal* **332**, 447–475 (1988).

6. Aschwanden, M. J. & Benz, A. O. On the Electron-Cyclotron Maser Instability. II. Pulsations in the Quasi-stationary State. *Astrophysical Journal* **332**, 466 (1988).

7. Fleishman, G. D., Stepanov, A. V. & Yurovsky, Y. F. Radio signature of fragmented electron injection into a coronal loop. *Solar Physics* **153**, 403–417 (1994).

8. Melrose, D. B. & Sy, W. N. Plasma emission processes in a magnetoactive plasma. *Australian Journal of Physics* **25**, 387 (1972).

9. Kuijpers, J. A Coherent Radiation Mechanism for Type IV dm Radio Bursts. *Solar Physics* **36**, 157–169 (1974).

10. Melrose, D. B. & Stenhouse, J. E. Emission and absorption of Langmuir waves by anisotropic unmagnetized particles. *Australian Journal of Physics* **30**, 481–493 (1977).

11. Zaitsev, V. V. & Stepanov, A. V. The plasma radiation of flare kernels. *Solar Physics* **88**, 297–313 (1983).

12. Winglee, R. M. & Dulk, G. A. The electron-cyclotron maser instability as a source of plasma radiation. *Astrophysical Journal* **307**, 808–819 (1986).

13. Stepanov, A. V., Kliem, B., Krüger, A., Hildebrandt, J. & Garaimov, V. I. Second-Harmonic Plasma Radiation of Magnetically Trapped Electrons in Stellar Coronae. *Astrophysical Journal* **524**, 961–973 (1999).

14. Melrose, D. B., Dulk, G. A. & Smerd, S. F. The polarization of second harmonic plasma emission. *Astronomy & Astrophysics* **66**, 315–324 (1978).

15. Ledenev, V. G. The directivity and polarization of radio emission at the second harmonic of plasma frequency from coronal magnetic loops. *Astronomy & Astrophysics* **285**, 1019–1022 (1994).

16. Willes, A. J. & Melrose, D. B. The Polarisation of Second Harmonic Coronal Type III Bursts. *Solar Physics* **171**, 393–418 (1997).

17. Fleishman, G. D. & Yastrebov, S. G. Nonlinear treatment for solar radio spikes. 2: The fastest growing mode. *Solar Physics* **153**, 389–402 (1994).

18. Stupp, A. Electron-cyclotron maser observable modes. *Monthly Notices of the Royal Astronomical Society* **311**, 251–268 (2000). `astro-ph/9904119`.

19. Benz, A. O. & Kuijpers, J. Type IV DM bursts - Onset and sudden reductions. *Solar Physics* **46**, 275–290 (1976).
