# Supplementary material for: Loss-cone instability modulation due to a magnetohydrodynamic sausage mode oscillation in the solar corona
Source: Nat Commun. 2019 May 23;10:2276. doi: 10.1038/s41467-019-10204-1 (PMC6533292; doi:10.1038/s41467-019-10204-1)
Supplement: Supplementary file 3 — Description of Additional Supplementary Files [file 41467_2019_10204_MOESM3_ESM.docx]

**Description of Supplementary Files**

**File Name:** Supplementary Movie 1

**Description:** Supplementary Movie 1 shows the NRH pulsating radio source at 228 MHz overlaid on a PFSS extrapolation of the quiet AR magnetic field. Note the extrapolation is at a fixed time (12:04 UT), does not evolve and is plotted to illustrate the general position of the radio source in the active region. The top panel in the middle shows the pulsations in the Orfées dynamic spectrum, with a light curve extracted at pulsation maximum intensity at 208 MHz shown in the bottom panel. The small panel at upper right shows the flux density spectrum (arbitrary units) at the time indicated by the vertical line on the dynamic spectrum. As explained in the main article text, radio emission initially builds up at the footpoints of coronal loops at Q, revealing an initial injection of energetic electrons into the region from approximately 12:45 UT. When the pulsations begin in the dynamic spectra, the radio source shows a repeated brightening at point P to the north-east. At numerous stages the radio source displays a repeated north-east to south-west motion. This motion is likely only an apparent one due to alternating brightness of the sources at P and Q, see below.

**File Name:** Supplementary Movie 2

**Description:** Supplementary Movie 2 shows 20 seconds from the pulsation event, revealing each specific pulse in detail. On the left, we plot a HMI magnetogram with PFSS extrapolated field; overlaid on this is a three-color image of 228 (red), 298 (green) and 327 (blue) MHz. The dynamic spectrum shows a zoom of the pulsations, revealing the signatures of electron beams in the form of reverse drift bursts. The bottom panel shows the light-curves at the 208 (pulse maximum frequency), 298 and 327 MHz. While pulsing occurs at 228 MHz around point P, the sources from 228-327 MHz remain relatively stationary at Q.
